# Assessing cancer-related fatigue: Validation of the Korean version of the cancer fatigue scale among cancer survivors

**DOI:** 10.1016/j.apjon.2025.100657

**Published:** 2025-01-20

**Authors:** Haneul Lee, Eun Young Park, Kwang-Hi Park

**Affiliations:** a Department of Physical Therapy, College of Medical Science, Gachon University, Incheon, South Korea; b College of Nursing, Gachon University, Incheon, South Korea

**Keywords:** Fatigue, Neoplasms, Cancer survivors, Quality of life, Validation study

## Abstract

**Objective:**

This study aimed to validate the Korean version of the Cancer Fatigue Scale (CFS-K) as a reliable tool for assessing cancer-related fatigue (CRF) for cancer survivors.

**Methods:**

A total of 208 cancer survivors who completed active treatment participated in evaluating the reliability, construct validity, and factor structure of the CFS-K through confirmatory factor analysis (CFA). Correlations with the Functional Assessment of Chronic Illness Therapy-Fatigue (FACT-F) and the European Organization for Research and Treatment of Cancer Quality of Life Questionnaire Core-30 (EORTC QLQ-C30) scales were analyzed to assess construct validity.

**Results:**

The CFS-K demonstrated strong psychometric properties, with high internal consistency (Cronbach’s *α* ​= ​0.875) and CFA validated a three-factor structure (physical, cognitive, and affective fatigue) with acceptable model fit indices (normed χ^2^ ​= ​2.62, CFI ​= ​0.899, TLI ​= ​0.878, RMSEA ​= ​0.088, SRMR ​= ​0.069). The standardized factor loadings for all items exceeded 0.5. Construct validity was confirmed through strong correlations with FACT-F (*r* ​= ​0.43–0.73) and significant correlations with EORTC QLQ-C30 subscales. Cancer survivors reported significantly higher fatigue levels across all subscales than controls.

**Conclusions:**

The CFS-K is a reliable and valid tool for assessing multidimensional CRF in cancer survivors.

## Introduction

Approximately three-quarters of individuals who have undergone cancer treatment experience adverse health outcomes.^1^^,^^2^ Among these, fatigue emerges as one of the most prevalent and distressing symptoms, often persisting long after the conclusion of treatment. Fatigue, particularly in cancer survivors, significantly affects daily functioning and quality of life (QoL).^3^^,^^4^ Cancer-related fatigue (CRF) is a prevalent and challenging symptom that affects individuals in all stages of their cancer experience, including those who survived cancer.^5^ CRF is defined as “a distressing, persistent, subjective sense of tiredness or exhaustion related to cancer or cancer treatment that is not proportional to recent activity and interferes with usual functioning”.^6^ Fatigue is especially common among long-term survivors, with approximately 30% to 40% of cancer survivors experiencing persistent fatigue for years following their diagnosis.^5^^,^^7^ This fatigue not only affects various aspects of daily functioning but also reduces QoL and delays the patient’s return to normal life.^3^^,^^8^

Accurate assessment of CRF requires multidimensional tools that capture the complex nature of CRF, as it affects physical, emotional, and cognitive domains.^9^ Various tools, such as the Functional Assessment of Chronic Illness Therapy-Fatigue (FACT-F), the European Organization for Research and Treatment of Cancer Quality of Life Questionnaire (EORTC QLQ-C30), and the Multidimensional Fatigue Symptom Inventory (MFSI),^10^ have been developed to assess these multidimensional aspects of CRF. Each tool offers unique strengths, with differences in psychometric properties, administration methods, number of items, and targeted dimensions for cancer patients and survivors.^9^^,^^11^

The Cancer Fatigue Scale (CFS), uniquely designed to address CRF, captures physical, affective, and cognitive fatigue in a concise, 15-item format. Unlike other tools, CFS provides a focused and comprehensive assessment of fatigue itself, rather than viewing it as a secondary aspect of QoL. Its simplicity allows even highly fatigued cancer patients to complete the scale within minutes, reducing response burden while maintaining psychometric robustness.^12^ This scale has been validated across various cancer patient populations, including individuals at different stages of the disease, but has yet to be validated specifically for survivors, who may experience distinct patterns of fatigue.^9^

Furthermore, CFS provides direct insights into cancer-specific fatigue dimensions, which many existing tools do not fully address, as they often evaluate fatigue’s broader impacts or consider it within the context of overall QoL. A recent study translated and validated the Korean version of the CFS (CFS-K) for cancer patients, confirming its clinical utility as a reliable and practical tool for assessing the multidimensional aspects of CRF.^13^ Despite the availability of established tools, none explicitly measures the multidimensional and cancer-specific nature of fatigue as comprehensively as CFS does. Validating the CFS-K for cancer survivors is critical to ensuring its applicability and accuracy in addressing the unique fatigue experiences of this population.

However, the specific fatigue experiences of cancer survivors may differ from those of patients undergoing active treatment, potentially driven by long-term factors such as accumulated physical damage, chronic inflammation, and psychological stressors.^14^^,^^15^ This difference underscores the need to validate the CFS-K specifically for cancer survivors, who may experience fatigue differently in the long term. Therefore, this study aims to evaluate the reliability and validity of the CFS-K as a tool for assessing CRF among cancer survivors.

## Methods

### Participants

In total, 210 cancer survivors were recruited through convenience sampling. The study included cancer survivors aged ≥ 18 years who had been diagnosed with cancer and completed active treatment (surgery, standard chemotherapy, and radiation therapy) within the past 5 years.^16^ The participants were required to have moderate CRF, as indicated by a Fatigue Numeric Rating Scale (NRS) score of ≥ 4 out of 10^17^, and the ability to comprehend and complete the questionnaires.

Recruitment was conducted by distributing participation posters with a survey link in regional cancer survivor centers, patient support groups (internet community), and via email sent to individuals who had previously participated in studies by our research team and consented to future contact. The general characteristics of the participants are presented in Table 1.

**Table 1:** Participants' characteristics (*N* ​= ​208).

Variable	*n* (%)	Mean ​± ​SD
Age, years		50.3 ​± ​11.6
Sex		
Women	131 (63.0)	
Men	77 (37.0)	
Marital status		
Married	166 (79.8)	
Single	32 (15.4)	
Widowed	3 (1.4)	
Divorced	7 (3.4)	
Fatigue NRS, score (0–10)		5.0 ​± ​1.9
Type of cancer		
Breast cancer	106 (51.0)	
Thyroid cancer	20 (9.6)	
Stomach cancer	15 (7.2)	
Colorectal cancer	15 (7.2)	
Prostate cancer	11 (5.3)	
Lung cancer	10 (4.8)	
Renal cancer	7 (3.4)	
Appendiceal cancer	6 (2.9)	
Gynecological cancer	5 (2.4)	
Biliary cancer	4 (1.9)	
Liver cancer	3 (1.4)	
Others^a^	6 (2.9)	
Time since complete treatment, days		744.6 ​± ​665.2

SD, Standard deviation; NRS, Numeric Rating Scale.

a Other cancers included pancreatic cancer (*n* ​= ​2), hematologic cancer (*n* ​= ​2), tongue cancer (*n* ​= ​1), and sarcoma (*n* ​= ​1).

Healthy control data were also collected, matched by age and sex, through community centers and local organizations for supplementary reference. Healthy controls were defined as individuals without a current or past cancer diagnosis and any serious chronic illness impacting daily function. These controls provided a baseline for typical fatigue levels in a healthy population, helping to distinguish CRF from general fatigue unrelated to cancer or other severe health conditions. The primary analysis of this study focused on cancer survivors, while the healthy control data are included as a supplementary reference in Supplementary File 1. None of the participants were involved in the initial CFS-K validation study.^13^

The sample size estimation was guided by prior research by MacCallum et al.,^18^ which recommends 4 to 10 participants per variable, with at least 100 participants overall, to ensure stability in factor analysis. Consequently, a minimum of 150 participants was considered necessary for this study. Additionally, this sample size aligns with other literature recommendations, suggesting at least 200 participants for robust factor analysis outcomes.^19^

### Data collection

A total of 430 questionnaires were distributed via email with an online survey link or in printed format. Participants accessed the survey link through various channels, including email invitations, recruitment posters, or in-person visits to our study sites. Of these, 419 responses were returned: 210 from cancer survivors and 209 from healthy controls, resulting in a response rate of 97.4%. Two responses from cancer survivors were excluded from the analysis due to incomplete responses, defined as participants who started but did not complete more than 30% of the questionnaire items. This resulted in a total of 417 questionnaires (208 from cancer survivors and 209 from healthy controls) for analysis. The study primarily focuses on analyzing the data from cancer survivors, while the data from healthy controls are included as a comparative reference. The average time for questionnaire completion was approximately 12 minutes, with a range of 10–15 minutes. Before beginning the survey, participants were presented with an electronic consent form embedded within the survey link, where they indicated consent. The questionnaire included demographic questions (age, sex, marital status, and cancer history for survivors), and participants provided self-reported medical information and CRF details.

### Scale revision process

An additional expert review process was conducted to ensure that the CFS-K is suitable for its intended purpose and appropriate for cancer survivors. The expert panel included three oncology nurse specialists, one psychology expert, and two professors from the rehabilitation department, who evaluated whether each item accurately reflects the fatigue experiences of cancer survivors. These experts reviewed the translated scale and determined that each item was appropriate for capturing the unique fatigue experiences specific to cancer survivors.

To establish the content validity of the questionnaire, the expert panel conducted a preliminary evaluation using the item-level content validity index (I-CVI).^20^ I-CVI was calculated by asking experts to rate each item on a four-point scale for both importance and feasibility. Items with an I-CVI of less than 0.8 were to be excluded from the final version; however, all items met the 0.8 threshold and were retained in the final scale. Details of the CFS-K are provided in Supplementary File 2.

### Measures

The CFS is an easy-to-use and validated tool designed to assess the multidimensional aspects of fatigue, including the physical, affective, and cognitive domains, in patients with cancer. In a previous study, the translation and adaptation of the CFS into Korean followed a rigorous process, including forward and back-translation, expert review, and pilot testing, ensuring cultural relevance and maintaining the scale’s original validity. This process resulted in favorable reliability and validity for the CFS-K.^13^ The original version of the scale has good reliability,^12^ and the Korean version also exhibits robust reliability, with Cronbach’s alpha values of 0.91, 0.65, 0.83, and 0.81 for the physical, affective, cognitive, and total scales, respectively.^13^

The Functional Assessment of Chronic Illness Therapy—Fatigue (FACT-F) is widely used to evaluate the severity of fatigue and its impact on daily activities in patients with cancer over the past week. The FACT-F, including its Korean version, has been validated for reliability, demonstrating high internal consistency in cancer populations.^20^ Both the original FACT-F^20^ and its Korean version have shown strong reliability and validity as tools for measuring fatigue.^21^

The European Organization for Research and Treatment of Cancer Quality of Life Questionnaire Core-30 (EORTC QLQ-C30) is a comprehensive tool used to assess the QoL of patients with cancer. It includes various functional and symptom scales, including fatigue, and has been consistently validated in different cultural contexts, including the Korean population.^22^^,^^23^

### Statistical analysis

All data were analyzed using SPSS for Windows (version 23.0; IBM, Armonk, NY, USA) and RStudio (version 4.4.2; Posit, PBC, Boston, MA, USA) with the Lavanan package. Descriptive statistics were applied to summarize the general and medical characteristics, as well as the patients’ ratings of CRF severity. The internal consistency reliability of all 15 items in the CFS was evaluated using Cronbach’s *α* coefficient, with a value ​> ​0.7 deemed satisfactory.^24^

Confirmatory Factor Analysis (CFA) was conducted to validate the factor structure of the CFS-K specifically for cancer survivors. The factor structure was derived from prior research involving cancer patients,^13^ where exploratory factor analysis (EFA) was conducted to establish the initial structure. The CFA tested whether the previously identified three-factor structure (physical, cognitive, and affective fatigue) holds for cancer survivors. The CFA model fit was assessed using indices such as the Comparative Fit Index (CFI), Tucker–Lewis Index (TLI), Root Mean Square Error of Approximation (RMSEA), and Standardized Root Mean Square Residual (SRMR). The acceptable thresholds for the model fit indices were defined as follows: normed χ^2^ ​≤ ​3; CFI and TLI ≥ 0.90; RMSEA and SRMR ≤ 0.08.^25^

Spearman’s rank correlation coefficient was used to assess construct validity by examining the relationships between the CFS-K and the FACT-F, as well as the subscales of the EORTC QLQ-C30.

### Ethical considerations

The study included cancer survivors and healthy controls. It was conducted in accordance with the Declaration of Helsinki and was approved by the Gachon University Institutional Review Board (IRB No. 1044396-202201-HR-016-02). All participants received an explanation of the study’s purpose, and those who agreed to participate signed an informed consent form before beginning the survey.

## Results

### Item analysis

The reliability of all 15 CFS-K items was considered acceptable, with Cronbach’s alpha coefficients of 0.875. The corrected item–total correlation values ranged from 0.292 to 0.682, with most items exceeding the threshold of 0.3, indicating acceptable alignment with the overall construct of CRF. Item 14 (“Encourage yourself to do something”) exhibited a lower item–total correlation of 0.292, suggesting it may assess a slightly distinct aspect of fatigue (Table 2). Analysis of Cronbach’s alpha if an item were deleted showed minimal impact on the scale’s internal consistency, with values ranging from 0.860 to 0.882. Notably, removing item 14 would increase Cronbach’s alpha to 0.882, the highest observed increase, suggesting that this item has the lowest consistency with the overall scale (Table 2).

**Table 2:** Item analysis of the CFS-K (*N* ​= ​208).

Item	Mean (SD)	Item-total correlation	Cronbach’s *α* if an item was deleted
1 Easily tired	3.39 (0.96)	0.610	0.864
2 Having the urge to lie down	3.17 (1.11)	0.594	0.864
3 Exhausted	2.97 (1.08)	0.682	0.860
4 Careless	3.19 (1.03)	0.660	0.862
5 Energetic feeling	3.06 (0.90)	0.430	0.871
6 Heavy and tired	3.19 (1.03)	0.652	0.861
7 Errors while speaking	2.67 (1.14)	0.564	0.865
8 Interest in something	3.04 (1.05)	0.307	0.882
9 Fed up	3.08 (1.14)	0.652	0.861
10 Forgetful	3.01 (1.14)	0.555	0.866
11 Ability to concentrate	3.21 (0.99)	0.374	0.878
12 Reluctant	3.14 (1.06)	0.607	0.863
13 Thinking has become slower	3.12 (1.17)	0.605	0.863
14 Encourage yourself to do something	3.44 (0.98)	0.292	0.882
15 Fatigue – you do not know what to do with yourself	2.75 (1.07)	0.624	0.863

SD, Standard deviation; CFS-K, Korean version of the Cancer Fatigue Scale.

### Confirmatory factor analysis

The CFA was conducted to validate the proposed three-factor structure of the CFS-K among cancer survivors. The model fit indices indicated an adequate fit to the data: χ^2^ (87) ​= ​227.592, *P* ​< ​0.001; CFI ​= ​0.899; TLI ​= ​0.878; RMSEA ​= ​0.088, 90% CI [0.074, 0.102]; and SRMR ​= ​0.069. The normed χ^2^ value was calculated as 2.62, which falls within the acceptable range of 2–3 for model fit. Although CFI and TLI were marginally below the acceptable threshold of 0.90, RMSEA and SRMR values were within acceptable ranges, indicating an overall acceptable fit.^26^ Table 3 presents the average variance extracted (AVE) values for each factor, which were 0.72, 0.68, and 0.71 for physical fatigue, cognitive fatigue, and affective fatigue, respectively. These AVE values exceed the square of the correlation coefficients between factors, supporting discriminant validity for the three-factor structure.

**Table 3:** Effect of each path of confirmatory factor analysis for the CFS-K (*N* ​= ​208).

	Items	Standardized Regression Weight	SE	CR (*t*-value)	*P* value	Construct Reliability	Average Variance Extracted	Cronbach’s *α*
F1	CFS1	0.715	0.105	9.240	< 0.001	0.92	0.72	0.889
	CFS2	0.824	0.106	10.886	< 0.001			
	CFS3	0.853	0.100	11.939	< 0.001			
	CFS4	0.673	0.089	10.593	< 0.001			
	CFS6	0.763	0.098	10.756	< 0.001			
	CFS9	0.778	0.110	9.856	< 0.001			
	CFS15	0.692	0.105	9.240	< 0.001			
F2	CFS7	0.672	0.091	10.597	< 0.001	0.91	0.68	0.826
	CFS10	0.656	0.093	10.597	< 0.001			
	CFS12	0.633	0.089	8.923	< 0.001			
	CFS13	0.556	0.098	10.540	< 0.001			
F3	CFS5	0.866	0.137	7.125	< 0.001	0.88	0.71	0.725
	CFS8	0.839	0.138	7.975	< 0.001			
	CFS11	0.690	0.133	7.500	< 0.001			
	CFS14	0.892	0.136	6.630	< 0.001			
The goodness of fit indices								
χ^2^		Normed χ^2^	CFI	TLI	RMSEA	SRMR		
227.592	< 0.001	2.616	0.899	0.878	0.088	0.069		

CFS-K, Korean version of the Cancer Fatigue Scale; CFI, Comparative fit index; TLI, Tucker–Lewis index; RMSEA, Root mean square error of approximation; SRMR, standardized root mean square residual.

The standardized factor loadings for each item were as follows: the physical fatigue factor comprised seven items with loadings ranging from 0.69 to 0.82; the cognitive fatigue factor included four items with loadings ranging from 0.56 to 0.85; and the affective fatigue factor also included four items with loadings ranging from 0.65 to 0.89. All factor loadings exceeded 0.5, indicating that each item loaded well onto its respective factor, thereby confirming the internal consistency of each factor.

### Construct validity

Table 4 presents the correlation matrix for the three subscales and the total score of the CFS-K relative to related measures. The construct validity of the CFS-K was examined using two complementary approaches: criterion validity and hypothesis testing validity.

**Table 4:** Correlation of the CFS-K Subscales and total score with FACT-F and EORTC QLQ-C30 measures (*N* ​= ​208).

	CFS-K Physical fatigue	CFS-K Cognitive fatigue	CFS-K Affective fatigue	CFS-K Total
FACT-F	0.73	0.62	0.43	0.76
EORTC QLQ-C30				
Global health/QoL	−0.59	−0.41	−0.46	−0.61
Physical functioning	−0.62	−0.53	−0.41	−0.66
Role functioning	−0.52	−0.48	−0.29	−0.55
Emotional functioning	−0.61	−0.60	−0.33	−0.65
Cognitive functioning	−0.50	−0.63	−0.32	−0.60
Social functioning	−0.46	−0.42	−0.28	−0.49
Fatigue	0.69	0.50	0.37	0.68
Nausea and vomiting	0.39	0.35	0.20	0.40
Pain	0.42	0.38	0.23	0.44
Dyspnea	0.41	0.42	0.24	0.45
Insomnia	0.38	0.40	0.25	0.43
Appetite loss	0.37	0.38	0.25	0.41
Constipation	0.26	0.25	0.15	0.28
Diarrhea	0.28	0.23	0.11	0.26
Financial difficulties	0.32	0.33	0.16	0.35

CFS-K, Korean version of Cancer Fatigue Scale; EORTC QLQ-C30, European Organization for Research and Treatment of Cancer Quality of Life Questionnaire Core 30; FACT-F, Functional Assessment of Chronic Illness Therapy Fatigue.

Criterion validity was assessed by examining the correlations between the CFS-K and FACT-F scores, as both tools measure cancer-related fatigue. The total and subscale scores of the CFS-K exhibited significant correlations with fatigue and its effect on activities of daily living, as measured by FACT-F (*r* ​= ​0.43–0.73, *P* ​< ​0.001). The strongest correlation was observed between the FACT-F score and CFS-K physical fatigue subscale (*r* ​= ​0.73, *P* ​< ​0.001), confirming the alignment between these tools in measuring similar constructs of fatigue.

Hypothesis testing validity was assessed using the EORTC QLQ-C30 scores. The correlation coefficients between the CFS-K and various subscales of the EORTC QLQ-C30 demonstrated significant relationships across physical, emotional, and cognitive functioning, as well as symptoms such as fatigue, pain, and appetite loss (Table 4). The CFS-K physical and cognitive fatigue scores exhibited moderate to strong correlations with relevant subscales of the EORTC QLQ-C30, while the affective fatigue scores showed relatively weaker correlations.

### Reliability of the subscales

The reliability analysis of the subscales from the EFA revealed Cronbach’s alpha coefficients of 0.889 for the physical subscale, 0.826 for the cognitive subscale, and 0.742 for the affective fatigue subscale, demonstrating acceptable reliability for each dimension (Table 5). The mean inter-item correlations were 0.686 for physical fatigue, 0.652 for cognitive fatigue, and 0.536 for affective fatigue, with all corrected item–total correlations exceeding 0.3, confirming that each item was adequately aligned with its corresponding subscale. The range of item–total correlations varied from 0.504 to 0.770 across subscales, reflecting a consistent internal structure within each subscale.

**Table 5:** Reliability of CFS-K subscales (*N* ​= ​208).

Subscale	Number of items	Cronbach’ s *α*	Mean inter-item correlation	Range of item–total correlations
Physical fatigue	7	0.889	0.686	0.605–0.770
Cognitive fatigue	4	0.826	0.652	0.561–0.691
Affective fatigue	4	0.742	0.536	0.504–0.583

CFS-K, Korean version of Cancer Fatigue Scale.

## Discussion

This study aimed to validate the CFS-K in cancer survivors, confirming its reliability and validity for assessing CRF in this population. Given the persistent and multidimensional nature of CRF in survivors, oncology health care professionals need to be equipped with accurate tools like the CFS to assess fatigue comprehensively. This will help ensure that CRF is appropriately recognized and addressed throughout survivorship care.^13^

The study results demonstrate that the CFS-K, previously validated for patients with cancer, also exhibits strong psychometric qualities when applied to cancer survivors.^13^ The reliability results of this study align with those of previous studies, though the affective subscale showed slightly lower internal consistency. This difference may stem from the inherent diversity within the sample population, such as variations in age, education level, cancer type, and overall health, which could influence emotional responses. These factors likely contribute to the differences in interpretations and responses, particularly regarding emotional and psychological symptoms, highlighting the need for careful consideration of participants' characteristics in such assessments.

When assessing the construct validity of the CFS, the results showed moderate to strong positive correlations between the CFS and other recognized fatigue measures, including the FACT-F and the EORTC fatigue scales. Moreover, moderate negative correlations were observed between the CFS scores and the functional scales of the EORTC QLQ-C30, indicating that higher fatigue levels were associated with lower functional capacity. These findings align with the results of the original validation study^12^ and are consistent with those of previous research validating the CFS-K for cancer patients.^13^ The significant associations between the CFS scores and specific symptoms such as fatigue, pain, breathing difficulties, sleep issues, and loss of appetite, further support the validity of the CFS-K for cancer survivors. The convergence of these measures underscores the effectiveness of the CFS-K in capturing the multifaceted nature of CRF, which extends beyond physical tiredness to include psychological, emotional, and social dimensions.

A key significant finding of this study is that cancer survivors reported high levels of fatigue, indicating that CRF can persist even after completion of cancer treatment. The results emphasized that cancer survivors reported significantly higher levels of fatigue across all subscales (physical, cognitive, and affective) compared with healthy controls and even cancer patients from a previous study.^13^ This underscores the persistence and severity of CRF following treatment, suggesting that survivors continue to experience fatigue at higher levels than those undergoing treatment. Ongoing fatigue may result from a combination of factors including physical damage accumulated during treatment, chronic inflammation, and psychological stressors.^27^^,^^28^ These findings align with existing literature, which suggests that CRF among survivors involves not only physical fatigue but also substantial emotional and cognitive burdens that persist long after the conclusion of treatment.^7^

Another key point highlighted in this study is the demonstrated reliability of the CFS-K in measuring these multidimensional aspects of CRF. The CFS-K effectively assesses not only physical fatigue but also emotional and social factors, suggesting its utility in clinical settings for developing more systematic and comprehensive management strategies for cancer survivors. Integrating CFS assessments into routine clinical practice could significantly improve the overall QoL of cancer survivors by identifying those requiring targeted interventions. The ability of the CFS-K to reliably assess diverse aspects of fatigue suggests its potential as an effective tool in clinical settings. Regular assessments using the CFS can facilitate the early detection of chronic fatigue in cancer survivors and guide timely interventions aimed at improving physical and psychological well-being. The elevated levels of fatigue observed in this study highlight the need for ongoing monitoring and management of CRF during the survivorship phase.

### Implications for nursing practice and research

Incorporating the CFS into routine survivorship care allows health care providers to systematically track fatigue levels, identify those at greatest risk, and adjust care plans accordingly. Integrating the CFS-K into nursing practice promotes early detection and timely intervention, which is essential for alleviating CRF symptoms and improving the long-term well-being of survivors. The comprehensive nature of the CFS-K also emphasizes the importance of addressing not only the physical aspects of fatigue but also its emotional and cognitive dimensions, fostering a more holistic approach to patient care. This proactive approach can help mitigate the long-term impact of CRF on survivors' QoL, ultimately enhancing the overall care and support they receive.

### Limitations

Despite these strengths, a few limitations should be acknowledged. First, although a previous study included a detailed expert review process to adapt and translate the CFS-K for cancer patients, this study did not incorporate further precision methods specifically for cancer survivors. To capture the unique dimensions of fatigue experienced by survivors more comprehensively, future studies may benefit from employing a more systematic consensus method, such as Delphi or similar approaches, to enhance the rigor of expert review. Additionally, the cross-sectional design captures only a single time-point perspective, limiting insights into the persistence or progression of CRF over time. Future studies could benefit from a longitudinal approach to better capture changes in CRF and its associated factors. Finally, given the importance of aligning the definition and operationalization of fatigue in cancer care, qualitative approaches are recommended to explore CRF’s sub-domains, focusing on the perceptions and perspectives of cancer survivors.

## Conclusions

This study validated the CFS-K as a reliable and effective tool to assess CRF in cancer survivors. The results confirm its strong psychometric properties, making it a valuable instrument for understanding the multidimensional nature of fatigue in cancer survivors. While these findings suggest the CFS-K can enhance fatigue assessment, future research should explore its potential utility in clinical settings to monitor fatigue levels and support interventions aimed at improving survivors' QoL.

## CRediT authorship contribution statement

Haneul Lee: Conceptualization, data collection, formal analysis, investigation, and writing – original draft; Eun Young Park: Conceptualization, data collection, and writing – original draft; Kwang-Hi Park: Conceptualization, data collection, writing – original draft, and funding acquisition. All authors had full access to the study data, and the corresponding author had the final responsibility for deciding to submit the manuscript for publication. The corresponding author attests that all listed authors meet the authorship criteria and that no individuals meeting the criteria have been omitted.

## Ethics statement

This study was conducted in accordance with the guidelines of the Declaration of Helsinki and approved by the Institutional Review Board of Gachon University (IRB No. 1044396-202201-HR-016-02). All participants provided written informed consent.

## Funding

This study was supported by the “R&D” program for Forest Science Technology (Grant No. 2021393A00-2123-0103) provided by the 10.13039/501100003664Korea Forest Service (Korea Forestry Promotion Institute) and the 10.13039/501100003725National Research Foundation of Korea (NRF) grant funded by the Korean government (MSIP; Ministry of Science, ICT and Future Planning) (Grant No. RS-2023-00253569). The funders had no role in considering the study design or in the collection, analysis, interpretation of data, writing of the report, or decision to submit the article for publication.

## Data availability statement

The datasets generated in this study are available from the corresponding author upon request.

## Declaration of generative AI and AI-assisted technologies in the writing process

No AI tools/services were used during the preparation of this work.

## Declaration of competing interest

The authors declare no conflict of interest.
