# Supplementary material for: Assessing cancer-related fatigue: Validation of the Korean version of the cancer fatigue scale among cancer survivors
Source: Asia Pac J Oncol Nurs. 2025 Jan 20;12:100657. doi: 10.1016/j.apjon.2025.100657 (PMC11868950; doi:10.1016/j.apjon.2025.100657)
Supplement: Multimedia component 1 [file mmc1.docx]

**Supplementary 1.**

**STable 1.** Participant characteristics (n = 417)

| **Variable** | Cancer survivors  (n=208) | Healthy controls  (n=209) | *P* value |
| --- | --- | --- | --- |
| Age, years | 50.3 ± 11.6 | 50.5 ± 11.5 | 0.833 |
| Sex |  |  |  |
| Women | 131 (63.0) | 144 (68.9) | 0.216 |
| Men | 77 (37.0) | 65 (31.1) |  |
| Marital status |  |  |  |
| Married | 166 (79.8) | 168 (80.4) | 0.914 |
| Single | 32 (15.4) | 30 (14.4) |  |
| Widowed | 3 (1.4) | 2 (1.0) |  |
| Divorced | 7 (3.4) | 9 (4.3) |  |
| Fatigue NRS, score (0–10) | 5.0 ± 1.9 | 4.2 ± 1.8 | 0.175 |

**STable 2.** Reliability assessment based on Cronbach’s alpha and the adjusted Cronbach’s alpha if individual items were deleted (n = 417)

| Item | Cronbach’s alpha (α) if an item was deleted | |
| --- | --- | --- |
|  | Cancer survivors  (n=208) | Healthy controls  (n=209) |
| Cronbach’s alpha (α) | 0.875 | 0.755 |
| Cronbach’s alpha (α) if item deleted |  |  |
| 1 Easily tired | 0.864 | 0.713 |
| 2 Having the urge to lie down | 0.864 | 0.711 |
| 3 Exhausted | 0.860 | 0.706 |
| 4 Careless | 0.862 | 0.710 |
| 5 Energetic feeling | 0.871 | 0.805 |
| 6 Heavy and tired | 0.861 | 0.706 |
| 7 Errors while speaking | 0.865 | 0.718 |
| 8 Interest in something | 0.882 | 0.797 |
| 9 Fed up | 0.861 | 0.720 |
| 10 Forgetful | 0.866 | 0.730 |
| 11 Ability to concentrate | 0.878 | 0.792 |
| 12 Reluctant | 0.863 | 0.726 |
| 13 Thinking has become slower | 0.863 | 0.724 |
| 14 Encourage yourself to do something | 0.882 | 0.785 |
| 15 Fatigue – you do not know what to do with yourself | 0.863 | 0.723 |

**STable 3.** Comparisons of the K-CFS scores between the cancer survivors and healthy controls (n = 417)

| **Item number and content of CFS-K** | **Mean (SD)** | | *P* value |
| --- | --- | --- | --- |
|  | Cancer survivors  (n=208) | Healthy controls  (n=209) |  |
| **Physical fatigue** |  |  |  |
| 1 Easily tired | 3.39 (0.96) | 2.95 (1.04) | <0.001 |
| 2 Having the urge to lie down | 3.17 (1.11) | 2.91 (1.23) | 0.022 |
| 3 Exhausted | 2.97 (1.08) | 2.31 (1.12) | <0.001 |
| 4 Careless | 3.14 (0.92) | 2.47 (0.95) | <0.001 |
| 6 Heavy and tired | 3.19 (1.03) | 2.74 (1.09) | <0.001 |
| 9 Fed up | 3.08 (1.14) | 2.71 (1.09) | <0.001 |
| 15 Fatigue – you do not know  what to do with yourself | 2.75 (1.07) | 2.12 (0.99) | <0.001 |
| **Cognitive fatigue** |  |  |  |
| 7 Errors while speaking | 2.67 (1.14) | 2.43 (1.09) | 0.024 |
| 10 Forgetful | 3.01 (1.14) | 2.71 (1.09) | 0.007 |
| 12 Reluctant | 3.14 (1.06) | 2.86 (0.97) | 0.005 |
| 13 Thinking has become slower | 3.12 (1.17) | 2.67 (1.07) | <0.001 |
| **Affective fatigue** |  |  |  |
| 5 Energetic feeling | 3.06 (0.90) | 3.24 (0.96) | 0.042 |
| 8 Interest in something | 3.04 (1.05) | 3.39 (1.06) | 0.001 |
| 11 Ability to concentrate | 3.21 (0.99) | 3.37 (0.96) | <0.001 |
| 14 Encourage yourself to do something | 3.44 (0.98) | 3.88 (0.87) | <0.001 |
