# Supplementary material for: Assessing cancer-related fatigue: Validation of the Korean version of the cancer fatigue scale among cancer survivors
Source: Asia Pac J Oncol Nurs. 2025 Jan 20;12:100657. doi: 10.1016/j.apjon.2025.100657 (PMC11868950; doi:10.1016/j.apjon.2025.100657)
Supplement: Multimedia component 2 [file mmc2.docx]

**Supplementary 2. Korean version of the Cancer Fatigue Scale (CFS-K)**

다음의 설문은 당신이 경험할 수 있는 피로감에 대한 질문입니다. 각 질문에 대해 당신의 현재 상태를 가장 적절하게 설명하고 있는 번호 하나를 선택하여 ○ 표시를 해주십시오. 각 질문에 대해 깊이 생각하기 보다는 첫 느낌을 기준으로 대답하여 주십시오.

| 문항 | 전혀 그렇지 않다 | 거의 그렇지 않다 | 약간 그렇다 | 상당히 그렇다 | 매우 그렇다 |
| --- | --- | --- | --- | --- | --- |
| 1. 쉽게 피곤해지십니까? | 1 | 2 | 3 | 4 | 5 |
| 2. 누워있고 싶은 마음이 드십니까? | 1 | 2 | 3 | 4 | 5 |
| 3. 기운이 다 빠진 느낌이 드십니까? | 1 | 2 | 3 | 4 | 5 |
| 4, (매사에) 주의력이 떨어졌다고 느끼십니까? | 1 | 2 | 3 | 4 | 5 |
| 5. 활력이 있습니까? | 1 | 2 | 3 | 4 | 5 |
| 6. 몸이 무겁고 나른하다고 느끼십니까? | 1 | 2 | 3 | 4 | 5 |
| 7. 말할 때 실수가 늘어난 것 같다고 느끼십니까? | 1 | 2 | 3 | 4 | 5 |
| 8. 무엇인가에 흥미가 느껴 지십니까? | 1 | 2 | 3 | 4 | 5 |
| 9. (어떤 것을 할 때) 지쳐서 하기 싫다고 느끼십니까? | 1 | 2 | 3 | 4 | 5 |
| 10. 자주 깜박하거나 건망증이 있다고 느끼십니까? | 1 | 2 | 3 | 4 | 5 |
| 11. (어떤 것을 할 때) 집중할 수 있으십니까? | 1 | 2 | 3 | 4 | 5 |
| 12. (어떤 것을 할 때) 주저하게 되거나 귀찮다고 느끼십니까? | 1 | 2 | 3 | 4 | 5 |
| 13. 생각이 느려졌다고 느끼십니까? | 1 | 2 | 3 | 4 | 5 |
| 14. 어떤 것을 하기 위해서 스스로 자신을 격려할 수 있습니까? | 1 | 2 | 3 | 4 | 5 |
| 15. 너무 피곤해서 무엇을 해야 할 지 잘 모르겠다고 느끼십니까? | 1 | 2 | 3 | 4 | 5 |

**The calculation Method**

1. Add the number together in every factor

Factor 1 (physical subscale) = (items 1 + 2 + 3 + 4 + 6 + 9 + 15) - 7

Factor 2 (cognitive subscale) = (items 7 + 10 + 12 + 13) - 4

Factor 3 (affective subscale) = 20 – (items 5 + 8 + 11 + 14)

1. Add the factors together

**Subtractions adjust for 1 as a state of no fatigue*
